# Imiquimod induced vitiligo‐like lesions—A consequence of modified melanocyte function

**DOI:** 10.1002/iid3.543

**Published:** 2021-10-06

**Authors:** Haiyan Yu, Jianping Cen, Xiaoxia Lin, Hao Cheng, Oliver Seifert

**Affiliations:** ^1^ Department of Dermatology, Sir Run Run Shaw Hospital Zhejiang University Medical College Hangzhou Zhejiang China; ^2^ Division of Dermatology and Venereology Ryhov Hospital Jönköping Sweden; ^3^ Division of Cell Biology, Department of Biomedical and Clinical Sciences Linköping University Linköping Sweden

**Keywords:** apoptosis, autophagy, Imiquimod, melanocytes, TLR7, TLR9

## Abstract

**Introduction:**

Imiquimod plays an important role in the management of condyloma and premalignant lesions. Successively, an increase of hypopigmented lesions following imiquimod application has been reported. However, the mechanisms of imiquimod on melanocytes remain unclear. This study was designed to assess the effect of Imiquimod on the functions of melanocytes in vitro.

**Methods:**

Primary cultured melanocytes were isolated from normal control skin tissue. After incubation with imiquimod for 48 h in vitro, cell viability was analyzed by cell counting kit‐8 assay. Apoptosis was detected using the Annexin V‐fluorescein‐5‐isothiocyanate flow cytometry assay. Melanin content and tyrosinase activity in melanocytes were measured by colorimetric method and the modified dopachrome method. The production of inflammatory cytokine interleukin 8 (IL‐8), IL‐6, and soluble ICAM‐1 (soluble Intercellular Adhesion Molecule‐1[sICAM‐1]) in melanocytes were measured by enzyme‐linked immunosorbent assay (ELISA). Toll‐like receptor 7 (TLR7), toll‐like receptor 9 (TLR9) protein, and autophagy‐related proteins microtubule‐associated protein 1A/1B‐light chain 3 (LC3‐II), p62, mechanistic target of rapamycin (mTOR), and Atg5 were assessed using western blot analysis.

**Results:**

Imiquimod significantly inhibited the activity of tyrosinase activity and decreased melanin content in melanocytes and significantly increased apoptosis and IL‐6, IL‐8, and sICAM‐1 production in melanocytes. Moreover, the expression of TLR7 and TLR9 proteins were significantly increased, and the expression of mTOR, p62 protein were markedly decreased, but the expression of LC3II/I and Atg5 protein were significantly increased in melanocytes after incubating with imiquimod.

**Conclusions:**

This study shows that imiquimod directly inhibits melanogenesis and increases melanocyte apoptosis rates. These effects combined with the upregulation of TLR7 and TLR9 together with increased autophagy activity and inflammatory cytokines production, might be the main reasons leading to hypopigmented lesions after imiquimod application.

## Graphical abstract

Imiquimod induces melanogenesis, activates autophagy, increases apoptosis in melanocytes, and stimulates the secretion of proinflammatory cytokines interleukin 6 (IL‐6), IL‐8 and soluble ICAM‐1 (sICAM‐1) as well as the expression of Toll‐like receptor 7 (TLR7) and toll‐like receptor 9 (TLR9) in melanocytes. These results suggest a possible mechanism causing imiquimod induced hypopigmentation. Physicians should be aware of this possible side effect that could be irreversible and difficult for the patient to accept.

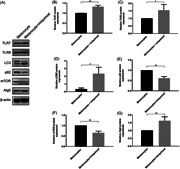

## 1 INTRODUCTION

Imiquimod cream modifies innate and acquired immune response by activating Toll‐like receptor 7 (TLR7) and cytokine production.^1^, ^2^ The antiviral, antitumor, and immunomodulatory properties of imiquimod are valuable to treat benign and malignant skin diseases as genital warts, actinic keratosis, and superficial basal cell carcinoma.^3^, ^4^ In line with more frequent clinical applications in dermatology^5^, ^6^ reports of hypopigmented lesions related to the use of imiquimod increased.^7^, ^8^, ^9^, ^10^, ^11^, ^12^, ^13^ A cytotoxic T‐cell‐mediated immune response has been suggested causing the imiquimod‐induced depigmentation,^14^ however, the exact mechanisms are still unclear.

Toll‐like receptors (TLRs) are well known pattern‐recognition receptors. They are important to the innate immune response by identifying pathogen‐associated molecular pattern (PAMPS), connecting innate and adaptive immune response.^15^, ^16^ Imiquimod as a TLR7 agonist can stimulate innate and adaptive immune response by inducing cytokines such as interferon‐alpha (IFN‐α), tumor necrosis factor‐alpha (TNF‐α), interleukin‐6 (IL‐6), and IL‐8.^17^, ^18^ It has been shown that human melanocytes express TLR2, 3, 4, 7, and 9,^19^, ^20^ and that the modulation of TLR expression affects the functions of melanocytes.^21^, ^22^ Kim et al.^23^ found that imiquimod induces human melanocytes apoptosis. Therefore, it can be speculated that imiquimod might directly act on the TLRs on melanocytes resulting in malfunction or destruction of melanocytes.

Autophagy is a lysosome‐dependent degradation pathway in eukaryotic cells. It is a major mechanism of preserving intracellular homeostasis and ensuring self‐protection. There is evidence underlining the role of autophagy in innate and acquired immunity^24^ and in melanocyte functions and melanin aggregation.^25^, ^26^, ^27^

This study was designed to assess the effect of imiquimod on melanocytes in vitro by analyzing melanogenesis, autophagy, apoptosis, and the expression of TLR7 and 9 and the production of inflammatory cytokines IL‐8, IL‐6, and soluble ICAM‐1 (soluble intercellular adhesion molecule‐1[sICAM‐1]).

## 2 MATERIALS AND METHODS

### 2.1 Primary epidermal melanocyte cultures

Primary epidermal melanocytes were obtained from three individuals undergoing routine circumcision at the Sir Run Run Shaw Hospital, Zhejiang University. For more details on the methods, please see Yu et al.^28^ Briefly, the primary melanocyte cultures were acquired as followed: epidermis was detached from dermis when incubated 12 h using a 0.25% Dispase solution (Roche Sigma‐Aldrich) at 4°C. The epidermis was processed using 0.25% trypsin solution for 10 min and then pipetted to disrupt cell clusters. The cellular suspension was cleaned through a 100  μm cell strainer and then centrifuged at 195* g* for 10 min to harvest the cells. Melanocytes were selectively grown in phorbol myristate acetate free melanocyte basal medium (ScienCell) with supplements and 100 µl/ml G 418 (Gibco life technologies) in a humidified incubator with 5% CO_2_ at 37°C. Cells from passages two to four were used for the experiments described. The imiquimod concentration (10 µg/ml) chosen in the experiments is based on the results of previous published data.^21^, ^23^

### 2.2 Analysis of cell proliferation

Cell proliferation was estimated by a colorimetric assay using CCK‐8 kit (Dojindo). Briefly, 5 × 10^3^ cells/well were cultivated in a 96‐well plate and incubated with or without imiquimod (10 μg/ml). After different timepoints of incubation, 10 μl CCK‐8 solution was added to the culture plate and cocultured with cells for 1 h in a CO_2_ incubator at 37°C. The optical density at 450 nm wavelength (OD450) was measured using a Microplate Reader (ThermoFisher Scientific).

### 2.3 Measurement of tyrosinase activity

We analyzed tyrosinase activity using the adapted dopachrome method. A total of 150 ml of 1 × 10^5^ cells/ml cultured melanocytes were placed in 96‐well plates. Forty‐eight hours cultivation was performed with or without imiquimod (10 μg/ml) in a moistened incubator with 5% CO_2_ at 37°C. The cells were then rinsed using phosphate‐buffered saline (PBS) and disrupted by 50 µl of 1% Triton X‐100/PBS. Afterwards, 100 ml of 1 mM l‐DOPA was placed in each well. Absorbance was the analyzed at 475 nm by the microplate reader after incubation at 37°C for 4 h.

### 2.4 Contents of melanin assay

A total of 1 × 10^5^ cultured melanocytes were cultivated in 12 well plates with or without imiquimod (10 μg/ml) for 48 h. Afterwards, we collected the melanocytes and measured the melanin amount in the cell suspensions using a colorimetric technique with 1 M NaOH. We then analyzed the relative melanin amount using a microplate reader at 400 nm absorbance.

### 2.5 Apoptosis studies

A total of 1 × 10^5^ melanocytes were cultured with or without imiquimod (10 μg/ml) in polystyrene 25 cm^2^ flasks (Grainer) for 48 h. We collected the melanocytes by a 0.25% trypsin solution lacking ethylenediaminetetraacetic acid (EDTA) and centrifuged the cells in cold PBS during 5 min at 1000 rpm and suspended in 300 µl of cold binding buffer (Immunotech). Apoptotic melanocytes were measured using Annexin V/PI (KeyGEN). Then flow cytometer analysis (Beckman Coulter) was performed following the fabricator's guidelines.

### 2.6 Analysis of IL‐6, IL‐8, and sICAM‐1 concentration

We analyzed the concentration of IL‐6, IL‐8, and sICAM‐1 using ELISA kits for the different melanocyte culture supernatants after 48 h with or without imiquimod (10 µg/ml). ELISA sets for IL‐6, IL‐8, and ICAM‐1 were applied (R&D Systems Europe Ltd.) according to the fabricator's guidelines. We showed the results in pg/ml (IL‐6, IL‐8) and in ng/ml for sICAM‐1.

### 2.7 Determination of protein concentration (western blot analysis)

We cultured 1 × 10^5^ melanocytes from control skin with or without imiquimod (10 μg/ml) for 48 h. Melanocytes were harvested and disrupted using radio‐immunoprecipitation assay buffer. We analyzed protein concentration in the lysates using bicinchoninic acid protein assay kit (Pierce Biotechnology). Proteins were detached using a 10% polyacrylamide gel, transported to a polyvinylidene difluoride membrane which were obstructed with skimmed milk for 2 h. Afterwards, we initiated incubation overnight using rabbit‐antibodies specific to TLR7 and toll‐like receptor 9 (TLR9) (Cell Signaling), microtubule‐associated protein 1A/1B‐light chain 3 (LC3) (Proteintech), p62 (Proteintech), mechanistic target of rapamycin (mTOR) (Abcam), Agt5 (Abcam), and β‐actin (Cell Signaling). After rinsing and incubation using Horseradish peroxidase‐conjugated secondary antibodies, we analyzed the signals with an ECL kit (Bio‐Rad). The densities of the band were calculated with a scanning densitometric analysis image software (National Institutes of Health).

### 2.8 Statistical analysis

Results were analyzed using the Student's *t* test in the GraphPad Prism 6 software (GraphPad Software Inc.). We showed our results as mean ± *SD. p* values below .05 were considered as statistical significant.

## 3 RESULTS

### 3.1 Imiquimod inhibits tyrosinase and decreases melanin content

**Figure 1:**
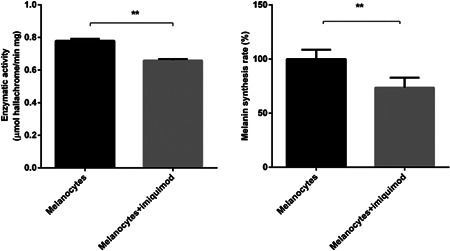
Imiquimod significantly inhibits tyrosinase activity and decrease the melanin content in primary cultured human melanocytes (*n* = 3, mean ± *SD*, **p < .01).

We measured the result of imiquimod incubation on the activity of tyrosinase as well as the effect of imiquimod on the amount of melanin in cultured human melanocytes. As shown in Figure 1, the amount of melanin and the activity of tyrosinase in melanocytes significantly diminished after incubation with imiquimod for 48 h (*p* < .01).

### 3.2 Imiquimod increases apoptosis

**Figure 2:**
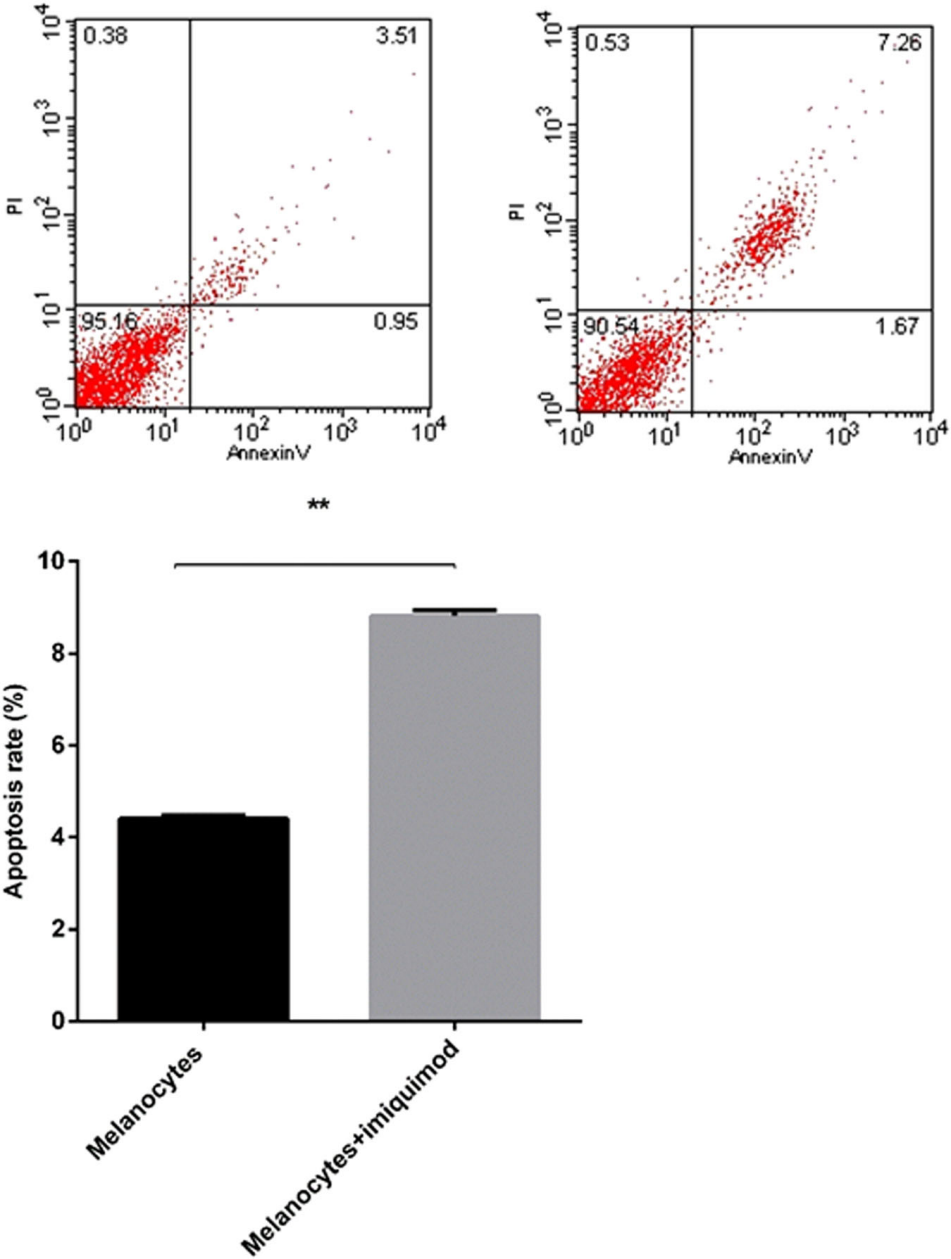
Flow cytometry assays show that imiquimod significantly increases the apoptosis rate of primary cultured human melanocytes (*n* = 3, mean ± *SD*, **p < .01).

A flow cytometry assay (Annexin V/PI) was used to analyze the degree of apoptosis in melanocytes. Our data revealed that melanocytes after incubation in imiquimod were significantly more prone to become apoptotic compared with cells cultured without imiquimod (Figure 2, *p* < .01).

### 3.3 Cytokine production amplified in melanocytes by imiquimod

**Figure 3:**
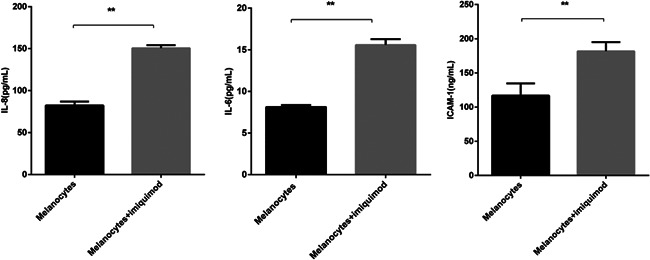
Imiquimod significantly increases the concentration of IL‐6, IL‐8, and sICAM‐1 in cell culture supernatants of melanocytes incubated for 48 h (*n* = 3, mean ± *SD*, **p < .01). IL‐6, interleukin 6; sICAM‐1, soluble ICAM‐1

The secretion of the inflammatory cytokines IL‐6, IL‐8, and sICAM‐1 were analyzed after 48 h in supernatants of melanocyte cultures. As shown in Figure 3, our data revealed significantly higher concentration of IL‐6, IL‐8, and sICAM‐1 in the supernatants of normal melanocytes incubated with imiquimod for 48 h (*p* < .01).

### 3.4 Imiquimod stimulates TLR7, TLR 9 expression, and activates autophagy in melanocytes

**Figure 4:**
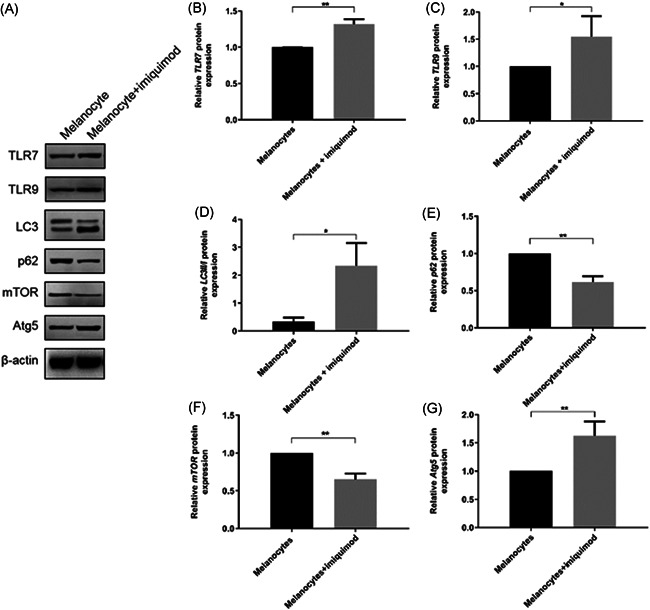
Imiquimod increases the expressions of TLR 7 (B), TLR9 (C) and of LC3II/I (D) and Atg5 (G) in melanocytes while p62 (E) and mTOR (F) expression significantly decreased (representative protein expression measured by Western blot (A), *n* = 3, mean ± *SD*; *p < .05, **p < .01). TLR7, Toll‐like receptor 7

TLR7 and TLR9 protein concentration in cells incubated with imiquimod for 48 h were significantly increased compared with melanocytes without imiquimod incubation (Figure 4). Furthermore, our data revealed that imiquimod incubation significantly increases the expression of LC3II/I and Atg5 protein in melanocytes but inhibits the expression of p62 protein and mTOR.

## 4 DISCUSSION

Previous studies have shown an important role of TLRs in melanocyte function. The stimulation of TLRs on melanocytes plays an important role in the production of pigment.^21^, ^22^, ^29^ Stimulation of TLR7 reduces melanogenesis of melanocytes,^29^ suppresses melanin synthesis and tyrosinase activity in melanocytes,^23^ elicit increased apoptotic rates,^23^ and leads to reduced numbers of melanocytes.^30^ In line with these findings, our results confirm that imiquimod may directly inhibit melanogenesis and induce apoptosis of melanocytes. Our data revealed that imiquimod upregulates TLR7 and TLR9 concentration which in turn may enhance the TLR7 signaling pathway resulting in the malfunction of melanocytes. Interestingly, we showed increased expression of TLR7 and TLR9 in vitiligo melanocytes in a recent study,^28^ emphasizing a role of TLR7 and TLR9 in hypopigmentation.

IL‐6 and IL‐8 have been proven to directly hinder melanocyte growth and lead to melanocyte programmed cell death.^31^, ^32^ Furthermore, higher expression of IL‐6 and IL‐8 have been found in vitiligo lesions.^33^ Stimulation of TLRs with TLR7 agonists induced the production of IL‐8 while the TLR9 ligand CpG 2006 induced the productions of IL‐6 and IL‐8 in melanocytes.^20^ This suggests that the activation of TLR7/TLR9 may induce IL‐6/IL‐8 resulting in decreased melanocytes proliferation and melanocyte apoptosis. sICAM‐1 can be expressed on melanocytes and it has been found in vitiligo lesions.^28^, ^34^, ^35^ In line with these findings, our results showed increased IL6/IL8 and sICAM‐1 concentration in melanocytes after incubation with imiquimod. This suggests that higher autocrine production of IL‐6, IL‐8, and sICAM‐1 in melanocytes could be an additional mechanism involved in the malfunction and reduced number of melanocytes associated with topical use of imiquimod.

Modulation of autophagy regulates the function of melanocytes.^36^ Regarding melanogenesis, activation of autophagy can have both, either a stimulating or inhibiting effect.^25^ Inducing autophagy by Rapamycin lead to a higher melanin index and induced tyrosinase, while LC3 knockdown with small interfering RNA (siRNA) reduced the amount of melanin and inhibited tyrosinase.^37^, ^38^ Deletion of autophagy‐ related genes as BECN1 can lead to the decline of melanin aggregation^39^ suggesting that promoting autophagy increase melanogenesis. On the contrary, resveratrol, an anti‐melanogenic substance, amplified autophagy and reduction of Atg5 inhibited resveratrol facilitated anti‐melanogenesis and autophagy in melanocytes.^40^ A light‐emitting diode (LED) with a wavelength of 585 nm can inhibit melanin production by inducing autophagy in melanocytes.^41^ The present data showed increased autophagy activity with significantly increased expression of LC3II/I and Atg5 protein and markedly decreased expressions of mTOR, p62 protein in melanocytes after incubating with imiquimod in vitro. Our data propose that upregulated autophagy leads to melanocyte malfunction associated with topical imiquimod treatment.

The present study suggest that imiquimod induces melanocyte apoptosis. This is in line with a previously published report^23^ and propose a possible mechanism in inducing vitiligo‐like lesions. However, this depigmentation occurs only in a limited number of treated patients. In conclusion, melanocyte apoptosis in imiquimod induced vitiligo‐like lesions has to be a multifactorial process as described for vitiligo.^42^ A cytotoxic T‐lymphocyte‐mediated autoimmune process^14^ as well as other forms of apoptosis such as necroptosis or pyroptosis might play an important role and need to be investigated in future studies.

A weakness of our study is the size of the study population. Nevertheless, the present study was intended to be a pilot study to comprehensively analyze for the first time the effect of imiquimod on a wide spectrum of melanocyte functions. In vivo experiments with increased number of participants and studies inhibiting TLRs would be of great interest to confirm the present data. Cultivation of cells and growth factors added to the cell cultures can change the phenotype and the functions of the examined cells. Therefore, the assumptions drawn from in vitro cell culture studies have to be cautiously interpreted. Still, since melanocytes stimulated or unstimulated, were cultivated under equal environments, the alterations seen between the cells are presumably not linked to cell‐culture settings. The time point of 48 h for the evaluation of the imiquimod effect can be debatable. Additional analysis at 24 h and after 72 and 96 h would have been of great interest and should be performed in future studies.

Current ongoing clinical studies and case reports implicate imiquimod as a new treatment option for skin melanoma metastasis^43^ and the results from our study may help to understand the pathomechanisms behind the effect of imiquimod on melanoma cells.

In conclusion, our data suggest possible novel pathomechanisms in what way topical imiquimod modifies different functions of melanocytes leading to the hypopigmented skin lesions. Imiquimod induces melanogenesis, activates autophagy, increases apoptosis in melanocytes, and induces secretion of proinflammatory mediators IL‐6, IL‐8, and sICAM‐1. Future studies have to confirm these results and address the question of TLR7 and TLR9 as a key player in mediating these effects.

## CONFLICT OF INTERESTS

The authors declare that there are no conflict of interests.

## ETHICS STATEMENT

The study protocol was approved by the ethics committee, Zhejiang University Medical College, China and the study conforms to the Declaration of Helsinki guidelines.

## AUTHOR CONTRIBUTIONS

Haiyan Yu, Xiaoxia Lin, Jianping Cen, Hao Cheng, and Oliver Seifert made substantial contributions to conception, design, analysis and interpretation of data. Haiyan Yu, Xiaoxia Lin, and Jianping Cen contributed to the acquisition of data. Haiyan Yu, Xiaoxia Lin, Jianping Cen, Hao Cheng, and Oliver Seifert have been involved in drafting the manuscript and revising it critically for important intellectual content. Haiyan Yu, Xiaoxia Lin, Jianping Cen, Hao Cheng, and Oliver Seifert gave their final approval of the version to be published. All authors agree to be accountable for all aspects of the work in ensuring that questions related to the accuracy or integrity of any part of the work are appropriately investigated and resolved.

## Data Availability

The data that support the findings of this study are available from the corresponding author upon reasonable request.
